# Treatment of nephrotic syndrome with anti-CD20 therapies in pregnancy: a case series and review of the literature

**DOI:** 10.1080/0886022X.2025.2481201

**Published:** 2025-03-27

**Authors:** Pierre-Alexis Gauci, Marion Cremoni, Jérôme Delotte, Flavia Vocila, Vincent L. M. Esnault, Barbara Seitz-Polski, Maxime Teisseyre

**Affiliations:** a Department of Obstetrics and Gynecology, Reproduction and Fetal Medicine, Nice University Hospital, Université Côte d’Azur, Nice, France; b Institut de Recherche sur le Cancer et Vieillissement UMR7284 CNRS INSERM U1081, Université Côte d’Azur, Nice, France; c Reference Center for Rare Diseases Idiopathic Nephrotic Syndrome and Membranous Nephropathy, Nice University Hospital, Université Côte d’Azur, Nice, France; d Department of Immunology, Nice University Hospital, Université Côte d’Azur, Nice, France; e Department of Nephrology, Dialysis and Transplantation, Nice University Hospital, Université Côte d’Azur, Nice, France; f Department of Nephrology and Dialysis, Cannes Hospital, Cannes, France

**Keywords:** Pregnancy, nephrotic syndrome, auto-immunity, rituximab, obinutuzumab

## Abstract

Membranous nephropathy (MN), focal segmental glomerulosclerosis (FSGS) and minimal change disease (MCD) are autoimmune kidney diseases and the most common causes of nephrotic syndrome. Anti-CD20 monoclonal antibodies are now recommended as first-line therapy for MN. Anti-CD20 monoclonal antibodies are also effective in steroid-dependent or frequently relapsing nephrotic syndrome associated with MCD or FSGS. Many women eligible for these treatments are of childbearing age. The impact of anti-CD20 therapies on pregnancy and fetal outcomes remains uncertain, particularly in glomerular diseases. We describe three cases of patients with glomerular disease treated with anti-CD20 therapies in the context of pregnancy and review the literature.

## Introduction

Membranous nephropathy (MN), focal segmental glomerulosclerosis (FSGS) and minimal change disease (MCD) are autoimmune kidney diseases and the most common causes of nephrotic syndrome. The recognition of these diseases as autoimmune has promoted the use of immunosuppressive drugs, such as anti-CD20 monoclonal antibodies. Rituximab is a chimeric immunoglobulin G (IgG) 1 monoclonal antibody that targets CD20 on B cells, resulting in B-cell depletion. Rituximab, is now recommended in MN as first line therapy [1]. Rituximab has also been shown to be effective in steroid-dependent or frequently relapsing nephrotic ­syndrome associated with MCD or FSGS [1]. Over time, a number of new anti-CD20 monoclonal antibodies have been developed, such as obinutuzumab and ofatumumab. Obinutuzumab is a humanized and glycoengineered anti-CD20 IgG1 monoclonal antibody. Modification of the glycan tree structure in the Fc region results in higher affinity for FcγRIII, thereby increasing antibody-dependent cellular cytotoxicity [2]. Preclinical data suggest that obinutuzumab is superior to rituximab in inducing B-cell depletion [2]. Obinutuzumab, has also been shown to be effective in MN and in steroid-dependent or frequently relapsing nephrotic syndrome [3–5]. With nearly two decades of use, the safety profile of rituximab is well known, with toxicities including infusion-related reactions and, less frequently, cytopenias and infections [6]. In hematological diseases, obinutuzumab has a similar safety profile but is generally associated with a higher incidence of adverse events than rituximab, some of which may be related to the higher cytokine release induced by this drug [6]. In patients with nephrotic syndrome, obinutuzumab was not associated with an increased incidence of adverse events compared to rituximab [5,7].

Many women eligible for these treatments are of childbearing age. There is very little data on the effects of anti-CD20 drugs during pregnancy in patients with nephrotic syndrome, as pregnancy is an exclusion criterion in many studies. Most data are from case series or retrospective studies of hematological or autoimmune neurological disorders [8–10]. Rituximab is unlikely to cross the placenta in the first trimester and no treatment-related malformations have been observed to date [8]. Transplacental transfer increases slowly during the second trimester, with fetal concentrations reaching maternal serum levels by 26 weeks’ gestation. Maximum IgG transfer occurs during the last 4 weeks of gestation, and fetal serum rituximab levels may exceed maternal levels at term [8]. The impact of anti-CD20 therapies on pregnancy and fetal outcomes remains uncertain, particularly in patients with nephrotic syndrome. Here, we describe three cases of patients with glomerular disease treated with anti-CD20 therapies in the context of pregnancy and we review the literature on the use of anti-CD20 drugs in pregnancy.

## Cases

### Case 1

**Figure 1.**
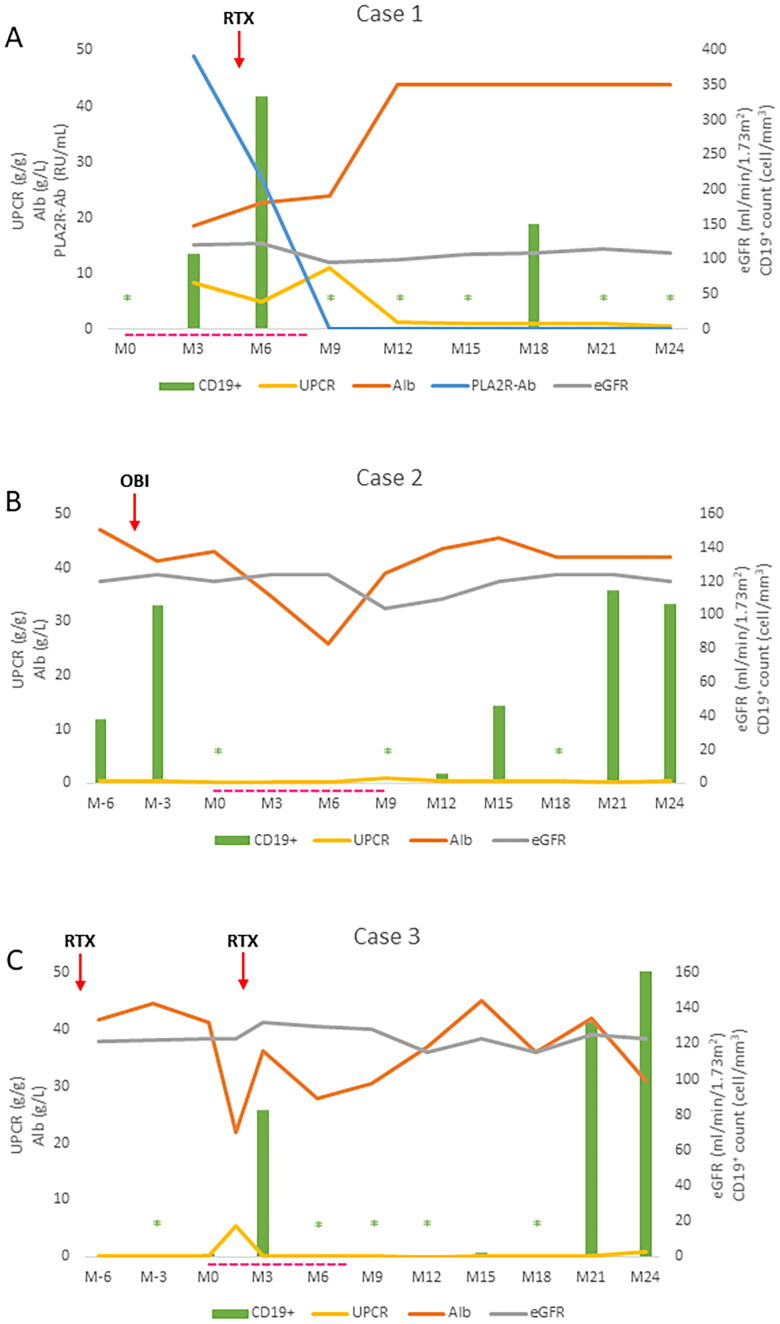
Trends in urine protein-creatinine ratio, serum albumin, estimated glomerular filtration rate, CD19^+^ count and anti-PLA2R antibody titer before, during and after pregnancy in the three cases. The pink dotted line represents the period of pregnancy. M0 indicates the beginning of pregnancy. For case 1, no biological data are available before pregnancy or at M0. The green star means that the CD19^+^ count is not available at this time.CD19^+^ cell count laboratory standards: 90–390/mm^3^. The anti-PLA2R antibody titer was determined by the ELISA test developed by EUROIMMUN (Medizinische Labordiagnostika AG, Lübeck, Germany). Abbreviations: Ab, antibody; eGFR, estimated glomerular filtration rate (CKD-EPI formula); OBI, obinutuzumab; PLA2R, phospholipase A2 receptor; RTX, rituximab; UPCR, urine protein-creatinine ratio.

A 32-year-old Caucasian woman was diagnosed with nephrotic syndrome at 14 weeks (week’s amenorrhea). At 16 weeks, kidney biopsy confirmed the diagnosis of stage 2–3 MN with interstitial fibrosis estimated at 10%. Anti-phospholipase A2 receptor (PLA2R) antibodies were positive with a titer of 48.8 RU/mL by enzyme-linked immunosorbent assay (ELISA). She received rituximab (1000 mg on days 0 and 15) at 20 weeks. Nephrological history is summarized in Table 1 and Figure 1A. She developed hypertensive disorders at 31 weeks, treated with labetalol (200 mg three times daily). A maturative corticosteroid therapy was administered (betamethasone two doses of 12 mg). Obstetrical ultrasound follow-up was normal. The patient remained hospitalized 30 days until induction of labor for worsening hypertensive disorders (repeated episodes of moderate high blood pressure associated with headache) at 35 weeks and five days. Due to failure of labor induction, a cesarean section was performed. Neonatal outcomes are reported in Table 2. Three months after treatment, the mother’s rituximab serum level was 7.1 µg/mL by ELISA. Immunological remission was achieved three months after rituximab (i.e., at 32 weeks’ amenorrhea) and clinical remission was achieved six months after rituximab (i.e., two months after delivery). Twelve months after delivery, she remains in immunological and partial clinical remission. The child is healthy and developing normally.

**Table 1.** Nephrological history.

Cases	Nephropathy	Age at diagnosis (years)	Time between diagnosis and pregnancy	Prior treatments	Treatments during pregnancy	Anti-CD20 treatment	Indication of anti-CD20 treatment	Time between anti-CD20 treatment and pregnancy
**Case 1**	pMN (PLA2R associated)	32	During pregnancy (16 w)	None	RTX	RTX	Severe active NS	During pregnancy (20 w)
**Case 2 **	MCD	23	6 years before pregnancy	CS, MMF, RTX, OBI	None	OBI*	Frequently relapsing and steroid-dependent NS	18 months (RTX) and 4 months (OBI) before pregnancy
**Case 3 **	MCD	29	18 months before pregnancy	CS, RTX	CS, RTX	RTX	Frequently relapsing and steroid-dependent NS	7 months before pregnancy and during pregnancy (9 w)

Abbreviations: CS, corticosteroids; MCD, minimal change disease; MMF, Mycophenolate mofetil; NS, nephrotic syndrome; PLA2R, Anti-Phospholipase A2 Receptor; pMN, primary membranous nephropathy; OBI, obinutuzumab; RTX, rituximab; w, weeks’ amenorrhea.

* Treatment with obinutuzumab due to the appearance of anti-rituximab antibodies. Anti-rituximab antibodies were detected by ELISA (LISA-TRACKER, Theradiag. Limit of detection defined by the manufacturer: 5 ng/mL).

**Table 2.** Neonatal outcomes.

Cases	Term of delivery	Delivery route	Newborn weight (g) / length (cm)	Apgar score at 1/5/10 min	Arterial cord pH / lactates (mmol/L)	Hospitalization unit and duration (days)
**Case 1**	35 w + 6 d	Cesarean	2230 / NA	10/10/10	7.25 / 1.9	Neonatology unit, 6 d
**Case 2 **	40 w + 1 d	Instrumental vaginal	2466 / 44	10/10/10	7.14 / 6.2	Maternity unit, 3 d
**Case 3 **	35 w + 3 d	Vaginal	2232 / 45	10/10/10	7.34 / 2.7	Maternity unit, 7 d

Abbreviations: d, day(s); NA, not available; w, weeks’ amenorrhea.

### Case 2

A 29-year-old North-African woman was diagnosed with MCD six years before pregnancy. She had one history of early miscarriage. Nephrological history is summarized in Table 1 and Figure 1B. Eighteen months prior to pregnancy, she received a course of rituximab (1000 mg on days 0 and 15) in addition to oral corticosteroid therapy (prednisone 1 mg/kg/day) to treat a relapse in this steroid-dependent and frequently relapsing patient, resulting in a complete remission. As anti-rituximab antibodies were detected at 74 ng/mL by ELISA, she received obinutuzumab four months before pregnancy (100 mg on day 0 and 900 mg on day 1) as maintenance treatment. The course of pregnancy was usual, without any fetal or maternal complication. Delivery was at term, with instrumental extraction due to fetal heart rate abnormalities. Neonatal outcomes are reported in Table 2. The neonatal hyperlactatemia associated with a pH below normal was considered to be acute hypoxia of respiratory and non-metabolic origin, as it was associated with fetal heart rhythm abnormalities associated with uterine contractions during delivery, explaining the decision for instrumental delivery [11]. Twelve months after delivery, she remains in complete clinical remission of nephrotic syndrome. The child is healthy and developing normally.

### Case 3

A 31-year-old North-African woman was diagnosed with MCD 18 months before pregnancy. She had one history of early miscarriage. Nephrological history is summarized in Table 1 and Figure 1C. Seven months prior to pregnancy, she received a course of rituximab (1000 mg on days 0 and 15) in addition to corticosteroid therapy (prednisone 1 mg/kg/day) to treat a relapse in this steroid-dependent patient who frequently relapsed, resulting in a complete remission. A new relapse of nephrotic syndrome occurred at seven weeks, with complete clinical remission achieved under corticosteroids (prednisone 1 mg/kg/day). She was treated with rituximab (1000 mg on days 0 and 15) at nine weeks. The dose of corticosteroid was gradually reduced during pregnancy. The course of pregnancy was usual until a premature rupture of membranes at 35 weeks. Moderate maternal hyperleukocytosis (13.6 10^9^/L) associated with a C-reactive protein of 10.9 mg/L was reported without microbiological documentation, and probabilistic antibiotic therapy was initiated. Labor started spontaneously in three days. Neonatal outcomes are reported in Table 2. The newborn experienced hypoglycemia with neonatal icterus and an asymptomatic cytomegalovirus infection without complications that did not require treatment. Twelve months after delivery, she remains in complete clinical remission of nephrotic syndrome. The child is healthy and developing normally.

## Discussion

During pregnancy, it is important to understand how to interpret proteinuria and determine its cause. Proteinuria is a main feature of preeclampsia but also a hallmark of glomerular disease. Preeclampsia resolves after delivery, while nephrotic syndrome may require specific immunosuppressive treatments. Proteinuria documented before pregnancy or before 20 weeks suggests a preexisting glomerular disease, whereas late onset proteinuria suggests preeclampsia. The soluble fms-like tyrosine kinase receptor-1 (sFlt-1)/placental growth factor (PlGF) ratio can be used to predict the risk of pre-eclampsia in women with a clinical suspicion of the syndrome [12]. Autoantibody testing may also be useful for the diagnosis of MN. However, this distinction is sometimes difficult and may require renal biopsy. Pregnancy is often considered a barrier to renal biopsy because of the risk of major complications, such as bleeding. A systematic review compared the outcome of 243 biopsies during pregnancy with 1236 biopsies after delivery [13]. Four cases of major hemorrhagic complications occurred in the 2nd trimester of pregnancy. Complications were more common during pregnancy than after delivery (7% *vs.* 1%, *p* = 0.001), with a possible peak at around 25 weeks’ gestation. Therefore, renal biopsy may be considered on a case-by-case basis after risk-benefit assessment, preferably before the 2nd trimester of pregnancy.

Pregnancy is known to modulate immune and renal function [14,15]. Hence, pregnancy is a potential window for the diagnosis of glomerular diseases. However, the diagnosis of nephrotic syndrome in pregnancy is rare, occurring in 0.012% to 0.025% of pregnant women [16–19]. In patients diagnosed with nephrotic syndrome prior to pregnancy, the relapse rate during pregnancy is not well established. In a case series of 18 pregnancies in patients with idiopathic nephrotic syndrome, relapse occurred in 5.5% of pregnancies [20]. This relapse rate was higher (60%) in a series of five pregnancies and occurred only in patients with frequent relapsing nephrotic syndrome prior to pregnancy [21]. Preexisting chronic kidney disease, hypertension and proteinuria are risk factors for pregnancy complications, such as maternal or perinatal death, prematurity or growth restriction [22,23]. The clinical presentation of these complications may mimic that of pre-eclampsia as in two of the reported cases [22,23]. Adverse outcomes are inversely correlated to renal function, and these risks are increased with concurrent proteinuria [24]. MN appears to have a better maternal and fetal prognosis than FSGS [19,25]. The two patients with MCD we describe had a history of miscarriage. Miscarriage is not uncommon in patients with nephrotic syndrome and is reported in up to 17.6% of pregnancies in MCD, 28.6% of pregnancies in FSGS and 40% of pregnancies in MN [19].

Rituximab is a first-line treatment for MN and a treatment for frequently relapsing or steroid-dependent nephrotic syndrome with proven safety and efficacy. Exposure to rituximab is reduced in nephrotic patients as compared with other rituximab-treated diseases, in part due to proteinuria resulting in urinary drug loss [26,27]. In MN, we have previously shown that serum rituximab levels were undetectable three months after rituximab infusion in 56% of patients, who were less likely to achieve clinical and immunological remission [28]. The risk of underdosing increases with the severity of nephrotic syndrome [28]. In idiopathic nephrotic syndrome, the prognostic impact of rituximab serum levels has not been studied. However, rituximab is most commonly administered after clinical remission following standard immunosuppressive treatment, including corticosteroids, which may improve its exposure compared to MN [29]. Pregnancy is a complex state in which changes in maternal physiology occur to support the development and growth of the placenta and the fetus. These adjustments can affect pharmacokinetics or pharmacodynamics, which determine the dosage and effect of drugs [30]. To our knowledge, there are no data on dose adjustment of anti-CD20 therapies during pregnancy. In our three patients, despite the pharmacological changes induced by pregnancy, anti-CD20 treatment systematically led to clinical remission.

Management of glomerular diseases may lead to exposure to anti-CD20 therapies before or during pregnancy, and little information is available in this context regarding maternal and neonatal outcomes. IgG, particularly IgG1, can cross the placenta using the neonatal Fc-receptor (FcRn) [31,32]. Cases of neonatal MN associated with transplacental transfer of IgG1 antibodies to neutral endopeptidase (NEP) have been described in NEP-deficient mothers immunized during a previous pregnancy [33]. Rituximab is an IgG1 monoclonal antibody and can therefore cross the placenta. It has been shown that if rituximab is administered in the 2nd or 3rd trimester of pregnancy, the newborn may have serum rituximab levels equal to or higher than the mother’s, resulting in B-cell depletion at birth [34,35]. However, the B depletion was shorter than in the mother, not complicated by infection or developmental abnormalities, and the vaccine response was normal [34,35]. The appearance of B cells correlated with the decrease in serum rituximab levels [34]. Therefore, CD19^+^ cell counts should always be checked in neonates to provide information on the safety and applicability of attenuated vaccines and the risk of induced immunosuppression. In our patients, anti-CD20 treatment had a favorable efficacy and safety profile. Case 3 presented with premature rupture of membranes. This has already been described in relation to exposure to rituximab during pregnancy or preconception [9,36]. We identified only three articles on rituximab exposure before or during pregnancy in nephrotic syndrome [37–39] (Table 3). No maternal or fetal adverse events were observed. In one case where rituximab was administered in the 2nd trimester of pregnancy, no neonatal B depletion was observed at birth [37]. Two case reports describe maintenance treatment with rituximab during pregnancy in patients with steroid-dependent or frequently relapsing nephrotic syndrome with a favorable maternal and fetal outcome [37,38]. More data are available for other diseases such as hematological malignancies and non-nephrotic autoimmune diseases. In 37 women with non-Hodgkin’s lymphoma treated with rituximab and concurrent chemotherapy during pregnancy, complete remission was achieved in 82% of cases. Two miscarriages and one fetal death occurred in woman treated during the 1st and 2nd trimesters. Six cases of respiratory/cardiac complications and five cases of hematologic abnormalities were reported in newborns [40]. In a previous study, 231 pregnancies associated with maternal exposure to rituximab were identified using the global rituximab safety database [8]. Indications for the mother included hematological malignancies, autoimmune cytopenias and other autoimmune diseases (including systemic lupus erythematosus, rheumatoid arthritis, idiopathic thrombocytopenia purpura, thrombotic thrombocytopenia, and multiple sclerosis). Of the 153 pregnancies with known outcomes, 90 resulted in live births. Twenty-two newborns were born prematurely and one neonatal death occurred. Eleven newborns had hematological abnormalities (peripheral B-cell depletion, neutropenia, lymphopenia, thrombocytopenia and anemia), without infectious complications. Four neonatal infections were reported (fever, bronchiolitis, cytomegalovirus hepatitis and chorioamnionitis). Two congenital malformations were noted (a clubfoot in a twin and a heart defect). However, it is important to note that most patients received other potentially immunosuppressive and teratogenic drugs at the same time as rituximab. One maternal death occurred due to preexisting autoimmune thrombocytopenia. In a retrospective cohort of 74 pregnancies in 55 women treated with rituximab for multiple sclerosis, rituximab was also shown to be safe [9]. A systematic review identified 102 pregnancies with rituximab use within six months of conception in patients with multiple sclerosis and neuromyelitis optica spectrum disorders: 78 resulted in live births and 12 in spontaneous abortions. Among 54 live births with reported gestational age, 31 occurred at term (after 37 weeks) and two before 32 weeks. B-cell counts were decreased in 39% of neonates and normalized within six months. No major safety signal was observed [10]. To our knowledge, we describe the first case of a patient treated with obinutuzumab before pregnancy.

**Table 3.** Summary of cases of exposure to rituximab in nephrotic syndrome before or during pregnancy reported in the literature.

Cases	Nephropathy	Age at diagnosis (years)	Demographics	Time between diagnosis and pregnancy	Prior treatments	Anti-CD20 treatment	Timing of anti-CD20 treatment	Indication of anti-CD20 treatment	Maternal outcome	Newborn outcome
**Case 1**	pMN (PLA2R associated)	39	NA	During pregnancy	None	RTX (two 1000 mg infusions)^a^	1st trimester of pregnancy	Active NS	At 38 w hypertension (>190/110 mmHg), with signs of fetal distress (cesarean section at 38 w). Persistent postpartum nephrotic syndrome with new course of RTX	Healthy newborn
**Case 2 **	FSGS	9	Japanese	17 years before pregnancy	CS, CsA, MZR, RTX	RTX (single dose of 375 mg/m^2^ every 6 months)^b^	Three months before pregnancy (1st pregnancy) and first trimester of pregnancy (2nd pregnancy)	Steroid-dependent NS	Delivery at 38 w (1st pregnancy) and 39 w (2nd pregnancy). No maternal complications and sustained clinical remission of NS.	Healthy newborns with normal development and no history of serious infection.
**Case 3 **	MCD	3	Caucasian	25 years before pregnancy	CS, MMF, CsA, CYC, RTX	RTX (1000 mg or 500 mg)^a^	Three months before first pregnancy (RTX 1000 mg) and second trimester of 1st pregnancy (RTX 1000 mg) and second trimester of 2nd pregnancy (RTX 500 mg)	Steroid-dependent NS	Delivery at 41 w (1st pregnancy with cesarean section for failure to progress) and 39 w (2nd pregnancy). No maternal complications and sustained clinical remission of NS.	Healthy newborns. Neonatal lymphocytes were normal on day 1 in the 1st pregnancy and normal on day 3 in the 2nd pregnancy.

Abbreviations: CS, corticosteroids; CsA, cyclosporin; CYC, cyclophosphamide; FSGS, Focal segmental glomerulosclerosis; MCD, minimal change disease; MMF, Mycophenolate mofetil; MZR, mizoribine; NA, not available; NS, nephrotic syndrome; PLA2R, Anti-Phospholipase A2 Receptor; pMN, primary membranous nephropathy; OBI, obinutuzumab; RTX, rituximab; w, weeks’ amenorrhea.

^a^ Without other concomitant immunosuppressive treatment.

^b^ Combined with a low dose (8 mg) of prednisolone.

Recommendations for the use of rituximab during pregnancy and breastfeeding vary worldwide. In France, the reference center for teratogenic agents states that rituximab may be used during pregnancy in the absence of other therapeutic options. Rituximab may also be used during breast-feeding [41]. The United States Food and Drug Administration and the American College of Rheumatology recommends that patients who may become pregnant should consider using birth control during treatment and for 12 months after treatment is stopped. Breastfeeding is not recommended during treatment and for six months after the last rituximab treatment [42]. The British Society for Rheumatology guidelines state that the rituximab may be considered to manage severe maternal disease in pregnancy if no other pregnancy-compatible drugs are suitable and maternal treatment with rituximab is compatible with breast milk exposure [43]. Therefore, a case-by-case assessment of the use of rituximab during pregnancy is necessary. Due to its long plasma elimination half-life and increased transplacental transfer at the end of pregnancy, it is advisable to administer it as early as possible in pregnancy to limit fetal exposure and potential complications associated with immunosuppression [44].

This study has several limitations. Firstly, we describe a limited number of cases. However, nephrotic syndrome is a rare disease and diagnosis or treatment is most often performed outside pregnancy. Secondly, biological monitoring (proteinuria, serum albumin and glomerular filtration rate) of newborns is not available. However, this was not indicated in healthy newborns and would have caused unnecessary blood loss. Thirdly, CD19^+^ count and serum rituximab levels are not available for newborns. This would have allowed a more accurate assessment of the degree of immunosuppression in the newborns. Finally, it is impossible to know whether the complications that occurred in these pregnancies were increased or decreased by anti-CD20 treatment, although they did not result in significant maternal or neonatal morbidity. Multicenter studies with a larger number of patients are needed to obtain more robust data.

In conclusion, pregnancy is a period at risk for exacerbation of glomerular disease. It is important that patients with nephrotic syndrome are made aware of the maternal and fetal risks that may be associated with their condition. Furthermore, treatment with anti-CD20 agents before or during pregnancy requires special maternal-fetal monitoring, but does not appear to pose a major health risk to the mother or newborn.

## References

[1] Rovin BH, Adler SG, Barratt J, et al. Executive summary of the KDIGO 2021 guideline for the management of glomerular diseases. Kidney Int. 2021;100(4):753–779. doi: 10.1016/j.kint.2021.05.015. 34556300

[2] Patz M, Isaeva P, Forcob N, et al. Comparison of the in vitro effects of the anti-CD20 antibodies rituximab and GA101 on chronic lymphocytic leukaemia cells. Br J Haematol. 2011;152(3):295–306. doi: 10.1111/j.1365-2141.2010.08428.x. 21155758

[3] Teisseyre M, Cremoni M, Boyer-Suavet S, et al. Advances in the Management of Primary Membranous Nephropathy and Rituximab-Refractory Membranous Nephropathy. Front Immunol. 2022;13:859419. doi: 10.3389/fimmu.2022.859419. 35603210 PMC9114510

[4] Hu X, Zhang M, Xu J, et al. Comparison of obinutuzumab and rituximab for treating primary membranous nephropathy. Clin J Am Soc Nephrol. 2024;19(12):1594–1602. doi: 10.2215/CJN.0000000000000555. 39207845 PMC11637703

[5] Dossier C, Bonneric S, Baudouin V, et al. Obinutuzumab in frequently relapsing and steroid-dependent nephrotic syndrome in children. Clin J Am Soc Nephrol. 2023;18(12):1555–1562. doi: 10.2215/CJN.0000000000000288. 37678236 PMC10723910

[6] Freeman CL, Sehn LH. A tale of two antibodies: obinutuzumab versus rituximab. Br J Haematol. 2018;182(1):29–45. doi: 10.1111/bjh.15232. 29741753

[7] Teisseyre M, Allinovi M, Audard V, et al. Obinutuzumab and ofatumumab are more effective than rituximab in the treatment of membranous nephropathy patients with anti-rituximab antibodies. Kidney International Reports; 2024. doi: 10.1016/j.ekir.2024.12.012. PMC11993203 40225374

[8] Chakravarty EF, Murray ER, Kelman A, et al. Pregnancy outcomes after maternal exposure to rituximab. Blood. 2011;117(5):1499–1506. doi: 10.1182/blood-2010-07-295444. 21098742

[9] Smith JB, Hellwig K, Fink K, et al. Rituximab, MS, and pregnancy. Neurol Neuroimmunol Neuroinflamm. 2020;7(4):e734. May 1 doi: 10.1212/NXI.0000000000000734. 32358226 PMC7217660

[10] Das G, Damotte V, Gelfand JM, et al. Rituximab before and during pregnancy: a systematic review, and a case series in MS and NMOSD. Neurol Neuroimmunol Neuroinflamm. 2018;5(3):e453. doi: 10.1212/NXI.0000000000000453. 29564373 PMC5858951

[11] Gauci PA, Racinet C, Ouellet P, et al. Eucapnic pH coupled with arterial cord pH improves hypoxic–ischemic encephalopathy prediction. Int J Gynaecol Obstet. 2024;165(3):1114–1121. doi: 10.1002/ijgo.15350. 38193307

[12] Zeisler H, Llurba E, Chantraine F, et al. Predictive value of the sFlt-1: plGF ratio in women with suspected preeclampsia. N Engl J Med. 2016;374(1):13–22. doi: 10.1056/NEJMoa1414838. 26735990

[13] Piccoli GB, Daidola G, Attini R, et al. Kidney biopsy in pregnancy: evidence for counselling? A systematic narrative review. BJOG. 2013;120(4):412–427. doi: 10.1111/1471-0528.12111. 23320849

[14] Mor G, Cardenas I. The immune system in pregnancy: a unique complexity. Am J Reprod Immunol. 2010;63(6):425–433. doi: 10.1111/j.1600-0897.2010.00836.x. 20367629 PMC3025805

[15] Hussein W, Lafayette RA. Renal function in normal and disordered pregnancy. Curr Opin Nephrol Hypertens. 2014;23(1):46–53. doi: 10.1097/01.mnh.0000436545.94132.52. 24247824 PMC4117802

[16] Gonzalez Suarez ML, Kattah A, Grande JP, et al. Renal disorders in pregnancy: core curriculum 2019. Am J Kidney Dis. 2019;73(1):119–130. doi: 10.1053/j.ajkd.2018.06.006. 30122546 PMC6309641

[17] Horigome M, Kobayashi R, Hanaoka M, et al. A case of minimal change nephrotic syndrome with pregnancy. CEN Case Rep. 2021;10(3):315–319. doi: 10.1007/s13730-020-00568-5. 33405175 PMC8271083

[18] Sato H, Asami Y, Shiro R, et al. Steroid pulse therapy for de novo minimal change disease during pregnancy. Am J Case Rep. 2017;18:418–421. doi: 10.12659/ajcr.902910. 28416778 PMC5404478

[19] Siligato R, Gembillo G, Cernaro V, et al. Maternal and fetal outcomes of pregnancy in nephrotic syndrome due to primary glomerulonephritis. Front Med (Lausanne). 2020;7:563094. doi: 10.3389/fmed.2020.563094. 33363180 PMC7758435

[20] Makker SP, Heymann W. Pregnancy in patients who have had the idiopathic nephrotic syndrome in childhood. J Pediatr. 1972;81(6):1140–1144. doi: 10.1016/s0022-3476(72)80246-4. 4643033

[21] Motoyama O, Iitaka K. Pregnancy in 4 women with childhood-onset steroid-sensitive nephrotic syndrome. CEN Case Rep. 2014;3(1):63–67. doi: 10.1007/s13730-013-0087-9. 28509244 PMC5411539

[22] Al Khalaf S, Bodunde E, Maher GM, et al. Chronic kidney disease and adverse pregnancy outcomes: a systematic review and meta-analysis. Am J Obstet Gynecol. 2022;226(5):656–670.e32. May doi: 10.1016/j.ajog.2021.10.037. 34736915

[23] Kendrick J, Sharma S, Holmen J, et al. Kidney disease and maternal and fetal outcomes in pregnancy. Am J Kidney Dis. 2015;66(1):55–59. Jul doi: 10.1053/j.ajkd.2014.11.019. 25600490 PMC4485539

[24] Tangren J, Bathini L, Jeyakumar N, et al. Pre-pregnancy eGFR and the risk of adverse maternal and fetal outcomes: a population-based study. J Am Soc Nephrol. 2023;34(4):656–667. Apr 1 doi: 10.1681/ASN.0000000000000053. 36735377 PMC10103349

[25] O’Shaughnessy MM, Jobson MA, Sims K, et al. Pregnancy outcomes in patients with glomerular disease attending a single academic center in North Carolina. Am J Nephrol. 2017;45(5):442–451. doi: 10.1159/000471894. 28445873

[26] Boyer-Suavet S, Andreani M, Cremoni M, et al. Rituximab bioavailability in primary membranous nephropathy. Nephrol Dial Transplant. 2019;34(8):1423–1425. doi: 10.1093/ndt/gfz041. 30929012

[27] Hartinger JM, Šíma M, Hrušková Z, et al. A novel dosing approach for rituximab in glomerular diseases based on a population pharmacokinetic analysis. Biomed Pharmacother. 2024;175:116655. doi: 10.1016/j.biopha.2024.116655. 38678967

[28] Teisseyre M, Cremoni M, Boyer-Suavet S, et al. Rituximab immunomonitoring predicts remission in membranous nephropathy. Front Immunol. 2021;12:738788. doi: 10.3389/fimmu.2021.738788. 34721403 PMC8548826

[29] Iijima K, Sako M, Nozu K, et al. Rituximab for childhood-onset, complicated, frequently relapsing nephrotic syndrome or steroid-dependent nephrotic syndrome: a multicentre, double-blind, randomised, placebo-controlled trial. Lancet. 2014;384(9950):1273–1281. doi: 10.1016/S0140-6736(14)60541-9. 24965823

[30] Feghali M, Venkataramanan R, Caritis S. Pharmacokinetics of drugs in pregnancy. Semin Perinatol. 2015;39(7):512–519. doi: 10.1053/j.semperi.2015.08.003. 26452316 PMC4809631

[31] Hyrich KL, Verstappen SMM. Biologic therapies and pregnancy: the story so far. Rheumatology. 2014;53(8):1377–1385. doi: 10.1093/rheumatology/ket409. 24352337

[32] Palmeira P, Quinello C, Silveira-Lessa AL, et al. IgG placental transfer in healthy and pathological pregnancies. Clin Dev Immunol. 2012;2012:985646–985613. doi: 10.1155/2012/985646. 22235228 PMC3251916

[33] Ronco P, Beck L, Debiec H, et al. Membranous nephropathy. Nat Rev Dis Primers. 2021;7(1):69. 34593809 10.1038/s41572-021-00303-z

[34] Decker M, Rothermundt C, Holländer G, et al. Rituximab plus CHOP for treatment of diffuse large B-cell lymphoma during second trimester of pregnancy. Lancet Oncol. 2006;7(8):693–694. Aug doi: 10.1016/S1470-2045(06)70797-5. 16887487

[35] Klink DT, van Elburg RM, Schreurs MWJ, et al. Rituximab administration in third trimester of pregnancy suppresses neonatal B-cell development. Clin Dev Immunol. 2008;2008:271363–271366. doi: 10.1155/2008/271363. 18596903 PMC2438602

[36] Perrotta K, Kiernan E, Bandoli G, et al. Pregnancy outcomes following maternal treatment with rituximab prior to or during pregnancy: a case series. Rheumatol Adv Pract. 2021;5(1):rkaa074. Jan 4 doi: 10.1093/rap/rkaa074. 33521513 PMC7819866

[37] Holden F, Bramham K, Clark K. Rituximab for the maintenance of minimal change nephropathy - a report of two pregnancies. Obstet Med. 2020;13(3):145–147. doi: 10.1177/1753495X18813739. 33093868 PMC7543165

[38] Nara M, Kaga H, Saito M, et al. Successful pregnancies in a patient with childhood-onset steroid-dependent nephrotic syndrome during rituximab maintenance therapy. Int Med. 2021;60(18):2985–2989. doi: 10.2169/internalmedicine.6633-20. PMC8502647 33776000

[39] Al-Rabadi L, Ayalon R, Bonegio RG, et al. Pregnancy in a patient with primary membranous nephropathy and circulating anti-PLA2R antibodies: a case report. Am J Kidney Dis. 2016;67(5):775–778. doi: 10.1053/j.ajkd.2015.10.031. 26744127 PMC4837089

[40] On S, Chang A. Treatment of lymphoma with rituximab and chemotherapy during pregnancy. Leuk Lymphoma. 2022;63(12):2897–2904. doi: 10.1080/10428194.2022.2100368. 35856478

[41] Crat L. Rituximab – Grossesse – Le CRAT; 2024 [Internet]. [cited 2025 Jan 22]. Available from: https://www.lecrat.fr/10810/.

[42] United States Food and Drug Administration. Rituxan final labeling text. [Internet]. Available from: https://www.accessdata.fda.gov/drugsatfda_docs/label/2021/103705s5467lbl.pdf.

[43] Russell MD, Dey M, Flint J, et al. British Society for Rheumatology guideline on prescribing drugs in pregnancy and breastfeeding: immunomodulatory anti-rheumatic drugs and corticosteroids. Rheumatology (Oxford). 2023;62(4):e48–e88. doi: 10.1093/rheumatology/keac551. 36318966 PMC10070073

[44] Li J, Levi M, Charoin J-E, et al. Rituximab exhibits a long half-life based on a population pharmacokinetic analysis in non-Hodgkin’s lymphoma (NHL) patients. Blood. 2007;110(11):2371–2371. Nov 16doi: 10.1182/blood.V110.11.2371.2371.17515402

